# Anilin: MAK-Begründung, Nachtrag

**DOI:** 10.34865/mb6253d10_1ad

**Published:** 2025-03-31

**Authors:** Andrea Hartwig, MAK Commission

**Affiliations:** 1 Institut für Angewandte Biowissenschaften. Abteilung Lebensmittelchemie und Toxikologie. Karlsruher Institut für Technologie (KIT) Adenauerring 20a, Geb. 50.41 76131 Karlsruhe Deutschland; 2 Ständige Senatskommission zur Prüfung gesundheitsschädlicher Arbeitsstoffe. Deutsche Forschungsgemeinschaft, Kennedyallee 40, 53175 Bonn, Deutschland. Weitere Informationen: Ständige Senatskommission zur Prüfung gesundheitsschädlicher Arbeitsstoffe | DFG

**Keywords:** Anilin, Hämatotoxizität, Methämoglobin, Entwicklungstoxizität, MAK-Wert, maximale Arbeitsplatzkonzentration, Schwangerschaftsgruppe

## Abstract

The German Senate Commission for the Investigation of Health Hazards of Chemical Compounds in the Work Area (MAK Commission) summarized and re-evaluated the data for the developmental toxicity of aniline [62-53-3]. Relevant studies were identified from a literature search. An occupational exposure limit value (maximum concentration at the workplace, MAK value) of 2 ml/m^3^ was previously set for aniline to maintain methaemoglobin (MetHb) levels below 5%, above which adverse effects are expected. This addendum evaluates whether the MAK value also protects the unborn child. There are no studies available on the effects on newborns exposed to maternal MetHb levels of up to 5% in utero. MetHb levels of up to 2% are considered to be within the physiological range for pregnant women. Newborns are significantly more sensitive to MetHb formers than adults. MetHb reductase activity in erythrocytes is 5 to 10 times as high in rats and mice as in humans. Thus, as the reduction of MetHb is much slower in humans they are more sensitive to MetHb formers than rodents. Therefore, developmental toxicity should not be assessed using data from these species. A risk to the unborn child cannot be ruled out at the MAK value for aniline of 2 ml/m^3^ and aniline has therefore been assigned to Pregnancy Risk Group B. This is supported by the assignment of dichloromethane and carbon monoxide to Pregnancy Risk Group B at a CO-Hb value of 5%. It was not possible to establish a concentration in air that would allow aniline to be classified in group C because there are no data to derive an aniline concentration that would not increase the MetHb levels above the range of fluctuation. However, reference is made to the justification of the requirement for Pregnancy Risk Group C for an aniline concentration in urine.

**Table:** 

**MAK-Wert (1983)**	**2 ml/m^3^ (ppm) ≙ 7,7 mg/m^3^**
**Spitzenbegrenzung (2002)**	**Kategorie II, Überschreitungsfaktor 2**
	
**Hautresorption (1981)**	**H**
**Sensibilisierende Wirkung (2006)**	**Sh**
**Krebserzeugende Wirkung (2006)**	**Kategorie 4**
**Fruchtschädigende Wirkung (2024)**	**Gruppe B**
**Keimzellmutagene Wirkung**	**–**
	
**BAT-Wert (2015)**	**500 µg (nach Hydrolyse)/l Urin^a)^**
**BLW (2015)**	**100 µg (aus Hämoglobin-Konjugat freigesetzt)/l Blut **
	
**1 ml/m^3^ (ppm) ≙ 3,864 mg/m^3^**	**1 mg/m^3^ ≙ 0,259 ml/m^3^ (ppm)**

^a)^ Ableitung des BAT-Wertes als Höchstwert wegen akut toxischer Effekte

Hinweis: Der Stoff kann gleichzeitig als Dampf und Aerosol vorliegen.

Im vorliegenden Nachtrag zur fruchtschädigenden Wirkung wird geprüft, ob beim MAK-Wert von 2 ml/m^3^ die Methämoglobin (MetHb)-Bildung eine Gefährdung des Ungeborenen darstellt. Die Daten zur fruchtschädigenden Wirkung beim Tier sind ausführlich in den Nachträgen aus den Jahren 2007 (Greim 2007), 2012 (Hartwig 2012) und 2018 (Hartwig und MAK Commission 2018) beschrieben.

Angaben zur Hintergrundbelastung mit Anilin in der Allgemeinbevölkerung und bei schwangeren Frauen finden sich in der Begründung des BAT-Werts (Brinkmann et al. 2025).

## Hintergrundbelastung: MetHb

Die Hintergrundbelastung in der nicht exponierten Allgemeinbevölkerung in den USA stellt sich nach ACGIH (2006) wie folgt dar: Mittelwert: 0,78 %, Median: 0,85 %, Standardabweichung: 0,37 %, 95. Perzentil: 1,28 %. Als Obergrenze kann 1,5 % MetHb festgesetzt werden. Der physiologische Hintergrundbereich für MetHb-Gehalte in Deutschland wird im Mittel mit < 1 % angegeben. Die Erhöhung des MetHb-Gehaltes beim Menschen über den Wert von 1,5 % hinaus ist als Expositionsmarker für MetHb-Bildner anzusehen (Leng und Bolt 2008). 

### Hintergrundbelastung mit MetHb bei Frauen und Schwangeren

Die Hintergrundwerte von MetHb bei Frauen und Schwangeren sind in Tabelle 1 dargestellt. In einer Probandenstudie wurden neun Frauen und zehn Männer sechs Stunden lang gegen 2 ml Anilin/m^3^ exponiert. Die Originaldaten zur MetHb-Bildung der neun Frauen wurden der Kommission zur Verfügung gestellt und sind in Tabelle 2 dargestellt (Käfferlein 2023). Zu Beginn der Exposition betrug der Mittelwert für MetHb bei den neun Frauen 0,75 % (Bereich: 0,53–0,97 %). Zusätzlich wurden bei acht Kontrollpersonen fünf Tage lang die MetHb-Gehalte bestimmt. Der Mittelwert betrug 0,58 ± 0,15 % (Bereich 0,2–1 %) und das 95. Perzentil 0,8 %. Für diese Personen wurde der Schwankungsbereich innerhalb eines Tages mit 21 %, der innerhalb von fünf Tagen mit 29 % angegeben (Käfferlein et al. 2014).

In einer Longitudinalstudie wurde der Einfluss von nitrathaltigem Trinkwasser auf den MetHb-Gehalt bei bis zu 357 schwangeren Frauen untersucht. Die Mittelwerte lagen je nach Schwangerschaftswoche im Bereich von 0,39–0,74 % (Maximalwert 3,6 %). Hervorgehoben wird die Anzahl der Frauen mit einem MetHb-Gehalt > 2 % (in der Publikation von Wright et al. (1999) als physiologische Obergrenze angegeben), die mit zwei bis fünf Schwangeren jedoch sehr gering war. Der Mittelwert des MetHb-Gehaltes nahm während der Schwangerschaft ab, auch nach Berücksichtigung einiger Kovariaten. Diskutiert wird der MetHb-senkende Einfluss von Vitaminen. Ob es zu Komplikationen während der Schwangerschaft kam oder zu Effekten bei den Neugeborenen wird nicht beschrieben (Manassaram et al. 2010).

In einer Umweltstudie wurde der Zusammenhang zwischen der SO_2_-Umgebungskonzentration und den MetHb-Werten bei 260 schwangeren Frauen untersucht. Der Bereich der MetHb-Gehalte kann aus einer Abbildung mit 1,2 bis 2 g/l angegeben werden (Mohorovic 2003). Da keine detaillierten Ergebnisse der MetHb-Werte angegeben werden, wird die Studie für die Hintergrundbelastung nicht berücksichtigt.

**Tab.1:** Hintergrundwerte von MetHb

Kollektiv	MetHb [%]		Literatur
Land	Mittelwert ± SD	Bereich	
9 Frauen	0,75	0,53–0,97	Käfferlein et al. 2014
eine Frau	0,27	–	
eine Frau	0,03	–	
8 Kontrollpersonen (k. w. A.) (Nichtraucher, 5 Tage)	0,58 ± 0,15 0,8 (95. Perzentil)	0,2–1	
Deutschland			
schwangere Frauen:			Manassaram et al. 2010
357 Erstuntersuchung	0,74 ± 0,48	0,1–2,2, n = 2^a)^	
317 (20. Schwangerschaftswoche)	0,67 ± 0,52	0,1–3,6, n = 3^a)^	
316 (28. Schwangerschaftswoche)	0,58 ± 0,46	0,1–2,1, n = 2^a)^	
304 (36. Schwangerschaftswoche)	0,51 ± 0,46	0,1–2,2, n = 2^a)^	
300 (Tag der Geburt)	0,42 ± 0,47	0,1–2,3, n = 2^a)^	
295 (2–4 Wochen nach Geburt)	0,39 ± 0,51	0,1–3,0, n = 5^a)^	
USA			
10 schwangere Frauen mit komplikationsfreier Schwangerschaft	1,3 ± 0,9	0,4–2,8 n = 3^a)^	Tabacova et al. 1997
Bulgarien			

MetHb: Methämoglobin

^a)^ mit > 2 % MetHb

## Wirkungsmechanismus

### Unterschiedliche Empfindlichkeit bezüglich der MetHb-Bildung zwischen Mensch und Nagern, sowie zwischen Erwachsenen und Feten/Neugeborenen

Das fetale Hämoglobin ist leichter oxidierbar als das von erwachsenen Menschen. Die Aktivität der MetHb-Reduktase, die für die Regeneration des funktionstüchtigen Häms aus MetHb verantwortlich ist, zeigt deutliche Speziesunterschiede und ist altersabhängig (Klimmek et al. 1988; Power et al. 2007; Rockwood et al. 2003). Die Aktivität der NADH-abhängigen Reduktase ist in Erythrozyten von Ratten und Mäusen im Vergleich zu denen des Menschen 5- bzw. 10-mal höher (Hartwig und MAK Commission 2017). In einer Untersuchung an adulten Tieren und Neugeborenen verschiedener Spezies sowie beim Menschen (n = 3 Erwachsene, 2 Neugeborene) war die MetHb-Reduktase-Aktivität beim erwachsenen Menschen ca. doppelt so hoch wie bei den Neugeborenen. Bei Ratten (n = 3 adulte, 16 neugeborene) und Mäusen (n = 4 adulte, 8 neugeborene) waren die Aktivitäten bei den neugeborenen deutlich höher als bei adulten Tieren (Lo und Agar 1986). Beim Menschen reagieren aufgrund der niedrigeren MetHb-Reduktase-Aktivität daher Neugeborene auf MetHb-Bildner mit höheren MetHb-Spiegeln als Erwachsene (Power et al. 2007).

In Erythrozyten sind die Aktivitäten der Glutathionreduktase beim Menschen etwa 5,5-mal und die der Katalase etwa 2,5-mal so hoch wie bei Ratten (Hartwig und MAK Commission 2017).

### Polymorphismus der MetHb-Reduktase

Für die Reduktion von MetHb zu Hämoglobin ist die lösliche NAD(P)H-abhängige Cytochrom b5-Reduktase 3 (Gen: *CYB5R3*) im Zytoplasma verantwortlich, die in Erythrozyten exprimiert wird. Als Elektronenakzeptor ist das am endoplasmatischen Retikulum membrangebundene Cytochrom b5-Typ A (Gen: *CYB5A*) zusätzlich für die Reaktion notwendig (Percy und Lappin 2008). Bei der seltenen angeborenen MetHb-Ämie liegen Mutationen im *CYB5R3*-Gen vor. Bisher wurden mehr als 80 verschiedene krankheitsverursachende Varianten im *CYB5R3*-Gen berichtet. Basierend auf der Schwere des Enzymdefekts werden zwei verschiedene Phänotypen der autosomal rezessiv vererbbaren MetHb-Ämie unterschieden. Beim Typ 1 führt eine missense-Variante nur in den Erythrozyten zu einem instabilen Enzym. Als Symptom tritt eine von Geburt an bestehende Zyanose auf, die verbunden ist mit leichten Beschwerden, wie Kopfschmerzen, Mattigkeit und Belastungsdyspnoe und in der Regel gut toleriert wird. Es können MetHb-Spiegel bis 30 % auftreten. Die meisten Patienten sind symptomfrei. Der Typ 1-Phänotyp tritt mit einer Häufigkeit von 1:1000 in einigen isolierten Populationen auf. Beim Typ 2 kommt es zu einer niedrigen Expressionsrate oder niedrigen Aktivität der Cytochrom b5-Reduktase 3 in allen Geweben, was zu einem veränderten Lipidstoffwechsel und neurologischen Beeinträchtigungen führt. Der MetHb-Spiegel liegt im Bereich von 8 bis 40 %. Die Typ 2-MetHb-Ämie weist aufgrund von neurologischen Störungen und retardiertem Wachstum einen deutlich schwereren Krankheitsverlauf auf. Diese Effekte treten ab dem 9. Lebensmonat auf. Die Sterblichkeit bis zum Erwachsenenalter ist sehr hoch. Ein Mangel an CYB5R in bestimmten Bevölkerungsgruppen der USA oder Russland ist nachgewiesen (Iolascon et al. 2021).

Frühgeborene haben einen niedrigeren Spiegel an MetHb-Reduktase als Erwachsene, wodurch sie einem erhöhten Risiko für die Entwicklung einer MetHb-Toxizität ausgesetzt sind. Jedoch werden Frühgeborene mit dem MetHb-Bildner NO inhalativ behandelt, um chronischen Lungenerkrankungen vorzubeugen. In einer Studie an 127 Frühgeburten wurde eine Genotypisierung von fünf Einzel-Nukleotid-Polymorphismen (SNP) im *CYB5A*- und *CYB5R3*-Gen durchgeführt, um den Zusammenhang zwischen den unterschiedlichen Genvarianten mit dem MetHb-Spiegel zu untersuchen. Die Frühgeborenen wurden inhalativ mit einer Konzentration von 12,6 ml NO/m^3^ behandelt, um eine Lungeninfektion zu vermeiden. Das Zeitintervall zwischen dem Beginn der NO-Behandlung und der Messung des ersten MetHb-Wertes lag zwischen einer und 24 Stunden. Es wurde kein Anstieg der MetHb-Werte mit der Dauer der NO-Exposition beobachtet. Säuglinge, die heterozygot für den SNP rs916321 im *CYB5R3*-Gen waren, hatten statistisch signifikant niedrigere MetHb-Werte als die Homozygoten, auch nach Anpassung für den ersten MetHb-Wert, das Geschlecht, Mehrlingsschwangerschaft, Geburtsgewicht und Gestationsalter. Obwohl nur ein geringer Unterschied bei den MetHb-Werten zwischen den rs916321-Varianten gefunden wurde, könnte sich nach Aussage der Autoren bei Interaktionen mit anderen genetischen oder umweltbedingten Risikofaktoren eine größere Wirkung zeigen (Fuller et al. 2015). Es wird nicht angegeben, ob sich die Genvarianten auf die Funktion des Proteins ausgewirkt haben, was jedoch vor dem Hintergrund, dass die Punktmutationen in den Introns lagen, eher dagegenspricht. Da alle MetHb-Werte unter 1 % lagen, hatten weder die inhalierte NO-Konzentration noch die Genvarianten eine klinische Relevanz. Wie hoch die MetHb-Spiegel bei den Müttern waren, wurde nicht angegeben.

## Toxikokinetik und Metabolismus

Informationen zur Toxikokinetik von Anilin beim Tier sind ausführlich in den Nachträgen (Greim 2007; Hartwig und MAK Commission 2018) beschrieben.

In einer Probandenstudie wurden neun Frauen und zehn Männer sechs Stunden lang gegen 2 ml Anilin/m^3^ exponiert (Käfferlein et al. 2014). 

Aus den Originaldaten wurde Folgendes berechnet: Da die Probanden während eines Sechstels der Expositionszeit mit einem Atemminutenvolumen von 30 l/min auf einem Fahrradergometer belastet wurden, entspricht dies einem durchschnittlichen Atemvolumen von 12,5 l/min, wenn ein Ruheatemvolumen von 9 l/min zu Grunde geleget wird. Die Daten zeigen, dass nach sechsstündiger inhalativer Exposition gegen 2 ml/m^3^, hochgerechnet auf acht Stunden und unter Berücksichtigung des erhöhten Atemvolumens (21 l/min), es bei sieben von neun Frauen zu einer Überschreitung von 1,5 % MetHb kommen würde (Tabelle 2).

Die Konzentration von Anilin im Urin betrug bei den neun Frauen am Ende der Exposition im Mittel 150,2** **µg/l (Käfferlein 2024).

**Tab. 2:** MetHb-Gehalte [%] von 9 Probandinnen bei 6-stündiger Exposition gegen 2 ml Anilin/m^3^ (Käfferlein 2023)

Probandin^a)^	Zeitpunkt [h]											
	0	2	4	6	7	8	10	12	24	48	MetHb^c)^	MetHb^d)^
1 langsam	0,70	1,20	1,70	2,07	–	1,40	0,97	1,00	0,60	0,83	2,52	**3,76**
2 schnell	0,70	1,05	1,13	1,27	–	0,87	0,70	0,83	0,50	0,90	1,46	**1,97**
3 langsam	0,97	0,90	0,95	1,30	1,30	0,77	1,00	1,03	1,10	0,77	1,41	**1,71**
4 langsam	0,85	0,90	0,97	1,33	1,00	0,57	0,83	0,87	0,50	0,57	1,49	**1,93**
5 schnell	0,80	0,83	1,00	1,07	0,80	0,67	0,73	0,63	0,53	0,45	1,16	**1,40**
6 langsam	0,63	0,80	0,77	0,90	0,70	0,67	0,75	0,60	0,63	0,53	0,99	**1,23**
7 langsam	0,93	0,93	1,07	1,30	1,00	1,00	0,87	0,70	0,73	0,67	1,42	**1,75**
8 langsam	0,53	0,73	0,87	1,03	0,80	0,83	0,80	0,70	0,67	0,60	1,20	**1,65**
9 langsam	0,60	0,80	0,97	1,03	0,77	0,60	0,73	0,70	0,60	0,40	1,18	**1,57**
**MW**	**0,75**	**0,82**	**1,05**	**1,26**	**0,91**	**0,82**	**0,82**	**0,79**	**0,65**	**0,64**	**1,43**	**1,89**
**MW (Inkrement^b)^)**				**0,51**	**0,16**	**0,07**	**0,07**	**0,04**	**–0,1**	**–0,11**		

^a)^ Acetyliererstatus

^b)^ Inkrement: Differenz des MetHb-Gehalts zum Zeitpunkt 0 h

^c)^ linear hochgerechnet auf 8-Stunden-Exposition aus Inkrement 0–6 h

^d)^ erhöhtes Atemvolumen für das Inkrement berücksichtigt

MW: Mittelwert

### Halbwertszeit von MetHb

Bei Intoxikationen durch aromatische Amino- oder Nitroverbindungen mit deutlich erhöhten MetHb-Werten beträgt deren Halbwertszeit bis zu zwölf Stunden (Bolyai et al. 1972; Leng und Bolt 2008).

Nach 6-stündiger inhalativer Exposition gegen 2** **ml Anilin/m^3^ wird für den Menschen eine Halbwertszeit (vermutlich terminal) von ca. 18 Stunden angegeben (Käfferlein et al. 2014). Aus der Abbildung 3 der Publikation geht jedoch hervor, dass die Halbwertszeit von MetHb ca. drei bis vier Stunden beträgt, wobei die initiale Halbwertszeit mit nur ca. zwei Stunden angegeben werden kann. Eine Akkumulation von MetHb über die Arbeitswoche ist damit nicht zu erwarten.

Die Halbwertszeit des MetHbs beträgt nach inhalativer Exposition gegen Anilin bei der Ratte 75 Minuten (Greim 2007). An trächtigen Ratten wurde gezeigt, dass Anilin die Plazentaschranke passieren kann. Innerhalb einer Stunde nach einmaliger subkutaner Verabreichung an die Muttertiere (1,3** **mg Anilin/kg KG) befand sich mehr Anilin im fetalen als im mütterlichen Plasma. Die Halbwertszeit im Blutplasma war mit 1,4 Stunden bei den Muttertieren und 1,5 Stunden bei den Feten in der gleichen Größenordnung Maickel und Snodgrass (1973).

## Entwicklungstoxizität

### Mensch

Zur Bewertung der fruchtschädigenden Wirkung wird beim Menschen die MetHb-Bildung durch Anilin als empfindlichster Endpunkt angesehen.

Bei 61 schwangeren Frauen wurden die MetHb-Werte bestimmt. Diese waren unabhängig vom Alter der Mutter, vom Gestationsalter und vom Raucherstatus. Die MetHb-Werte lagen bei den zehn Frauen mit normal verlaufender Schwangerschaft zwischen 0,4 und 2,8 %, bei 3 davon (30 %) über der von den Autoren als physiologisch angesehenen Grenze von 2 %. Insgesamt waren 64 % der MetHb-Werte höher als 2 %. Komplikationen in der Schwangerschaft wie Anämie (< 10** **g Hämoglobin/dl), vorzeitige Entbindung, Proteinurie, Ödeme, Bluthochdruck und Präeklampsie wurden gleichzeitig mit erhöhten MetHb-Werten bis zu 11,2 % beobachtet. Bei den betroffenen Müttern wurde eine Abnahme des reduzierten Glutathions und ein Anstieg des oxidierten Glutathions sowie der Lipidperoxide festgestellt. Die Autoren führen aus, dass die erhöhten MetHb-Werte auf eine hohe Exposition gegen Nitrit und Nitrat, sowie weitere MetHb-Bildner hinweisen. Für die Effekte werden Zellschädigungen, erhöhte Lipidperoxide und eine Abnahme der antioxidativen Reserven der Mütter verantwortlich gemacht (Tabacova et al. 1997).

In einer weiteren Studie dieser Arbeitsgruppe wurde bei 51 Mutter-Kind-Paaren am Tag der Geburt im Blut der Mütter und im Nabelschnurblut der MetHb-Gehalt, Glutathion, reduziertes Glutathion, Lipidperoxidase- und Peroxidase-Aktivität bestimmt. Anlass der Studie waren hohe Konzentrationen an NO_2_ in der Umgebungsluft (Mittelwert 23,1** **µg/m^3^, Spitzenkonzentrationen bis 238,5** **µg/m^3^) und hohe Konzentrationen an Nitrat im Trinkwasser (Trinkwasserversorgung: 8–54** **mg/l, Brunnenwasser: 13–400** **mg/l) und Gemüse (ca. 81 bis 1215** **mg/kg). Die MetHb-Mittelwerte waren im maternalen Blut 3,6 % und im Nabelschnurblut 6,9 %. Bei 3 von 42 Proben lag der Anteil an MetHb im Nabelschnurblut über 20 % mit einem Maximalwert von 34 %. Zwei dieser Fälle waren Frühgeburten, die wiederbelebt werden mussten. Mütter mit physiologisch „normalen“ MetHb-Konzentrationen von bis zu 2 % im Blut oder 2,8 % im Nabelschnurblut wurden in die „niedrige“, die anderen in die „hohe“ Gruppe eingeteilt. Weiterhin erfolgte eine Auswertung nach normalem Geburtsgewicht, niedrigem Geburtsgewicht (< 2500** **g), Geburt vor der 37. Schwangerschaftswoche und fetaler Notlage (Steißlage, Kaiserschnittgeburt). Der Anteil an Raucherinnen betrug 37 %, acht Mütter hatten während der Schwangerschaft eine Anämie, ob es sich dabei um Raucherinnen handelte, ist nicht angegeben. Bei 36 Müttern verlief die Schwangerschaft ohne Komplikationen mit normalen Geburtsgewichten, bei sechs Müttern trat eine Frühgeburt auf, bei sieben Neugeborenen war trotz normaler Schwangerschaftsdauer das Geburtsgewicht niedrig. 55 % der MetHb-Werte im mütterlichen Blut waren oberhalb von 2 % und 47 % der Nabelschnurblutwerte über 2,8 %. Die MetHb-Werte der Mütter mit Frühgeburten und mit fetaler Notlage waren etwa doppelt so hoch wie bei Müttern mit normaler Geburt. Die mittleren MetHb-Werte im Nabelschnurblut waren bei Frühgeburten viermal so hoch wie bei Normalgeburten, mit einem Höchstwert von 34 %. Der mittlere MetHb-Wert bei Neugeborenen mit niedrigem Geburtsgewicht war etwa 1,5-mal so hoch wie bei Neugeborenen mit normalem Geburtsgewicht, und der höchste gemessene Wert lag bei 16 %. Rauchen während der Schwangerschaft war weder im mütterlichen noch im Nabelschnurblut mit erhöhten MetHb-Werten assoziiert, obwohl das mittlere Geburtsgewicht der Neugeborenen von Raucherinnen niedriger war als bei denjenigen von Nichtraucherinnen (3046 g gegenüber 3197** **g). Raucherinnen hatten auch einen höheren Anteil an Frühgeburten (12 % gegenüber 6 %). Ergebnisse zum oxidativen Stress sind in Tabelle 3 dargestellt (Tabacova et al. 1998).

**Tab. 3:** Daten zum oxidativen Stress bei 51 Mutter-Kind-Paaren

Studie/Anzahl/Land	MetHb	Effekte	Literatur
51 Mutter-Kind-Paare, 37 % Raucherinnen, Bulgarien	normal/niedrig: bis 2 % im Blut der Mütter, bis 2,8 % im Nabelschnurblut, hoch: > 2 % (Blut der Mütter), > 2,8 % (Nabelschnurblut)	**mütterliches MetHb hoch**: tendenziell niedrigeres Geburtsgewicht, Blut: stat. sign.: Gesamt-GSH ↓, % GSH/Gesamt-GSH ↓, Lipidperoxidase ↑ (Blut u. Nabelschnurblut), **Frühgeburten**: MetHb (maternales Blut) doppelt so hoch, Nabelschnurblut bis zu viermal so hoch, Lipidperoxide ↑ (Nabelschnurblut), **niedriges Geburtsgewicht**: MetHb (maternales Blut) 1,5-mal so hoch (max: 16 %), Lipidperoxide ↑ (Nabelschnurblut)	Tabacova et al. 1998

GSH: Glutathion; max.: maximal; MetHb: Methämoglobin; stat. sign.: statistisch signifikant

### Tierexperimentelle Befunde

Seit den Nachträgen sind neue tierexperimentelle Studien publiziert worden.

Der Hauptmetabolit von Anilin ist N-Acetyl-4-aminophenol (Paracetamol). Paracetamol beeinflusst die fetale Entwicklung, insbesondere bei männlichen Feten, die Organogenese der Reproduktionsorgane und den Hormonstatus (Kristensen et al. 2011, 2012; Mazaud-Guittot et al. 2013). Die folgenden Studien überprüften bei Mäusen vergleichend die Wirkung von Anilin und Paracetamol nach Gabe während der Gestation.

Jeweils zehn trächtige C57BL/6JBomTac-Mäuse erhielten mit der Schlundsonde 50 oder 150** **mg Paracetamol/kg KG und Tag, sowie 31 oder 93** **mg Anilin/kg KG und Tag vom 7. Gestationstag bis zum Ende der Gestation. Bei den Muttertieren wurden keine Anzeichen von Toxizität festgestellt. Auch das Körpergewicht blieb unverändert. In allen Versuchsgruppen wurden keine Effekte auf Wurfgröße, Geschlechterverhältnis, Totgeburten und Wurfgewicht beobachtet. Bei allen Nachkommen wurde nach vier und sechs Wochen, bei acht Nachkommen jeder Gruppe nach acht und zehn Wochen der Anogenitalabstand gemessen. In der Gruppe der männlichen und weiblichen Nachkommen war nach Gabe der hohen Anilindosis von 93** **mg/kg KG der Anogenitalabstand zu allen Zeitpunkten statistisch signifikant vermindert, in der niedrigeren Dosisgruppe nur bei den weiblichen Nachkommen nach 6, 8 und 10 Wochen. Nur in der höchsten Paracetamoldosisgruppe war bei den männlichen Nachkommen und auch nur in der 10. Woche der Anogenitalabstand statistisch signifikant verringert. Bei den weiblichen Nachkommen war in der höchsten Paracetamoldosisgruppe zu allen Zeitpunkten und in der niedrigeren Dosisgruppe nur nach sechs Wochen der Anogenitalabstand statistisch signifikant verringert. Bei den sechs bis sieben Wochen alten männlichen und weiblichen Nachkommen wurden keine Effekte auf die Reproduktionsorgane beobachtet (Holm et al. 2015, 2016). Wie es Standard ist, wurde der Anogenitalabstand auf das Körpergewicht adjustiert, da das Körpergewicht als Confounder für diesen Parameter gilt. Die Geschlechtsunterschiede (Anogenitalabstand bei männlichen Ratten und Mäusen etwa doppelt so lang wie bei weiblichen Tieren) beruhen auf der Kontrolle durch Androgene und scheinen nicht durch Östrogene beeinflusst zu werden. Verringerter Anogenitalabstand bei männlichen Tieren und fehlender Effekt bei weiblichen Tieren deuten auf einen antiandrogenen Effekt hin, während erhöhter Anogenitalabstand bei weiblichen Tieren ohne Effekte bei männlichen Tieren auf einen androgenen Effekt hindeuten (US EPA 2005). Daher ist die Bedeutung des bei beiden Geschlechtern erniedrigten Anogenitalabstands in der Studie von Holm et al. (2015) unklar.

In einer weiteren Studie dieser Arbeitsgruppe erhielten jeweils zehn trächtige C57BL/6JBomTac-Mäuse mit der Schlundsonde 50 oder 150** **mg Paracetamol/kg KG und Tag, sowie 31 oder 90** **mg Anilin/kg KG und Tag vom 7. Gestationstag bis zum Ende der Gestation. Den Kontrolltieren wurde 0,5** **ml Trinkwasser per Schlundsonde appliziert. In der 8. Postnatalwoche wurde jeweils ein männliches Tier der acht Würfe der 150**-**mg-Paracetamol/kg-Dosisgruppe auf Urinmarkierung, Aggressivität und Sexualverhalten untersucht. In der 12.–13. Postnatalwoche wurde das Gehirn der männlichen Nachkommen seziert, um im sexuell dimorphen Nukleus (SDN) des präoptischen Areals (POA) im vorderen Hypothalamus Veränderungen zu untersuchen. Es wurde gezeigt, dass der SDN bei männlichen Nagetieren 2,5–7-mal größer ist als bei weiblichen Nagetieren, wobei der Unterschied von den induzierten Prostaglandin-E2-Spiegeln abhängt, die durch die Wirkung von Testosteron entstehen. Die durchschnittliche Anzahl der Calbindin-immunreaktiven Zellen (CALB-ir-Zellen) betrug 44 bei der Kontrollgruppe und 41 bei den männlichen Tieren der niedrigen Dosisgruppe von Paracetamol und war somit nicht statistisch signifikant unterschiedlich. Im Gegensatz dazu verringerte die Gabe von 150** **mg Paracetamol/kg KG und Tag die Zellzahl im SDN signifikant um etwa 50 % mit einer mittleren Zellzahl von 21. Der Rückgang der CALB-ir-Zellzahl, der bei den Anilin-exponierten männlichen Tieren beobachtet wurde, war ähnlich wie bei der höchsten Paracetamol-Dosis, wobei die höchste Anilin-Dosis einen statistisch signifikanten Rückgang auf eine mittlere Zellzahl von 21 ergab. Diese Ergebnisse deuten darauf hin, dass die intrauterine Exposition den Maskulinisierungsprozess des Gehirns beeinträchtigt haben könnte. Zur Urinmarkierung, Aggressivität und Sexualverhalten wurden nur die männlichen Tiere der hohen Paracetamol-Dosisgruppe untersucht. Die Tiere markierten ihren Käfig mit deutlich weniger Tropfen, dafür aber größeren. Die Aggressivität innerhalb von 30** **Minuten gegenüber einer fremden männlichen Maus nahm ab (statistisch signifikant verringerte Schnüffelvorgänge und Schwanzbewegungen (tail rattling), keine Bissattacken), sowie die sexuelle Aktivität (keine Vaginalpfropfen innerhalb von 30** **Minuten Verpaarungszeit) (Hay-Schmidt et al. 2017). Die untersuchten Parameter zur Charakterisierung des Verhaltens für Aggressivität sind nicht standardisiert und validiert. Es wurden nur acht Tiere untersucht.

## Bewertung

Als kritischer Effekt der Anilinwirkung beim Menschen wird die MetHb-Bildung und die Erythrozytentoxizität mit begleitenden Effekten auf die Milz bei der Ratte angesehen. Eine Erhöhung über 1,5 % zeigt eine Exposition gegen MetHb-Bildner an. Gesundheitsschädliche Effekte durch MetHb sind bei gesunden Erwachsenen bis zu einem MetHb-Gehalt von 5 % nicht zu erwarten (Leng und Bolt 2008).

**Fruchtschädigende Wirkung. **Untersuchungen zu Effekten bei Neugeborenen, die im Mutterleib gegen einen MetHb-Spiegel von bis zu 5 % exponiert waren, liegen nicht vor. MetHb-Spiegel bis zu 2 % bei Schwangeren werden als im physiologischen Bereich angesehen. Das fetale Hämoglobin wird leichter zu MetHb oxidiert als das von Erwachsenen. Weiterhin ist die MetHb-Reduktase-Aktivität bei Erwachsenen ungefähr doppelt so hoch wie bei Neugeborenen. Aufgrund beider Effekte reagieren Neugeborene deutlich empfindlicher als Erwachsene auf MetHb-Bildner. Die MetHb-Reduktase-Aktivität ist in Erythrozyten von Ratte und Maus um den Faktor 5 bis 10 höher als beim Menschen, somit reagiert der Mensch empfindlicher auf MetHb-Bildner als die beiden Spezies, da der Mensch das MetHb deutlich langsamer reduziert. Die Bewertung der entwicklungstoxischen Wirkung sollte daher nicht aufgrund von tierexperimentellen Daten erfolgen.

Bei Probandinnen betrug der von Käfferlein et al. (2014) gefundene individuelle MetHb-Höchstwert nach 6-stündiger inhalativer Exposition gegen 2 ml Anilin/m^3^ 2,07 % (langsame Acetyliererin). Unter Berücksichtigung des mittleren Basiswertes von 0,75 % MetHb betrug der Höchstwert des durch Anilin induzierten MetHb-Inkrements 1,32 %. Es wurde kein Plateau erreicht, so dass eine lineare Extrapolation auf acht Stunden erfolgen muss. Das Inkrement des Höchstwertes beträgt damit nach acht Stunden 1,8 % (1,32 % × 8/6). Bei linearer Umrechnung wäre das MetHb-Inkrement durch Anilin bei einem Atemminutenvolumen von 21 l/min (= 10 m^3^/8 Stunden) 3 % (1,8 % × 21 l/min/12,5 l/min). Es wird darauf hingewiesen, dass bei Schwangeren das Atemminutenvolumen um etwa 20–50 % nach Ende des ersten Trimenons u. a. durch die Wirkung des Progesterons zunimmt. Es liegt damit aber nicht höher als das angenommene Atemminutenvolumen von 21 l/min bei körperlicher Arbeit.

Da die NOAEC für den MetHb-Gehalt in Bezug auf die entwicklungstoxische Wirkung beim Menschen nicht bekannt ist und der Fetus deutlich empfindlicher reagiert als der Erwachsene, kann eine Gefährdung des Ungeborenen beim MAK-Wert von 2 ml Anilin/m^3^ nicht ausgeschlossen werden und deshalb erfolgt eine Zuordnung zu Schwangerschaftsgruppe B.

Unterstützend wird auf die Zuordnung von Dichlormethan und Kohlenmonoxid in Schwangerschaftsgruppe B in Bezug auf einen CO-Hb-Wert von 5 % hingewiesen (Weistenhöfer et al. 2023).

Es erfolgt keine Vergabe eines Hinweises auf Voraussetzung für Gruppe C für eine Anilinkonzentration in der Luft, da keine Daten vorliegen, aus denen sich eine Luftkonzentration ableiten lässt, die zu keiner Erhöhung der MetHb-Spiegel innerhalb der Schwankungsbreite führt. Jedoch wird auf die Begründung der Voraussetzung für Gruppe C für eine Anilinkonzentration im Urin hingewiesen (Brinkmann et al. 2025).
